# Exploring potential anti-inflammatory effects of medicinal cannabis

**DOI:** 10.1007/s00520-023-08069-8

**Published:** 2023-10-14

**Authors:** Taylan Gurgenci, Gregor Kijanka, Ristan Greer, Georgie Huggett, Phillip Good, Md Moniruzzaman, Janet Hardy

**Affiliations:** 1 Department Palliative and Supportive Care, 10th floor, Mater Health Services, Raymond Tce, South Brisbane, Queensland 4101 Australia; 2 Mater Research Institute, University of Queensland, Brisbane, Queensland Australia; 3 Queensland Micro- and Nanotechnology Centre, Griffith University, Nathan, Queensland Australia; 4 Torus Research, Brisbane, Queensland Australia; 5 Department of Palliative Care, St Vincent’s Private Hospital Brisbane, Brisbane, Queensland Australia; 6 School of Pharmacy, The University of Queensland, Brisbane, Australia

**Keywords:** Inflammation, Inflammatory cytokines, Medicinal cannabis, Advanced cancer

## Abstract

**Purpose:**

Inflammation is thought to play a key role in malignant disease and may play a significant part in the expression of cancer-related symptoms. Cannabidiol (CBD) is a bioactive compound in cannabis and is reported to have significant anti-inflammatory properties.

**Method:**

Serial C-reactive protein (CRP) levels were measured in all participants recruited to a randomised controlled trial of CBD versus placebo in patients with symptoms related to advanced cancer. A panel of inflammatory cytokines was measured over time in a subset of these patients.

**Results:**

There was no difference between the two arms in the trajectory of CRP or cytokine levels from baseline to day 28.

**Conclusion:**

We were unable to demonstrate an anti-inflammatory effect of CBD in cancer patients.

**Trial registration:**

ANZCTR 26180001220257, registered 20/07/2018.

**Supplementary Information:**

The online version contains supplementary material available at 10.1007/s00520-023-08069-8.

## Introduction

Inflammation plays an important role in tumour progression by either damaging resident tissues and facilitating tumorigenesis or fighting against cancer following an appropriate immune activation. Moreover, the positive association of chronic inflammation in the exacerbation of cancer is relevant to the discovery of new anti-cancer therapeutics. Consequently, the role of inflammation has been described as important in the aetiology, early detection, and prognosis of cancer [1–3].

Specific symptom clusters have also been found to be associated with inflammation [4]. Examples include the elevation of C-reactive protein (CRP) and tumour necrosis factor (TNF)-α in head and neck cancer pain [5], CRP and interleukin (IL)-1 receptor antagonist with fatigue during radiation therapy in breast and prostate cancer patients [6], and IL-6, soluble IL-6 receptor, soluble IL-1 receptors, IL-10, CRP, and macrophage inflammatory protein (MIP)-1α reflecting symptom burden in myeloma [7]. Fatigue and pain often co-exist in advanced cancer, and have been shown to have a significant association with the patient’s inflammatory state, especially with the levels of IL-6, IL-18, MCP-1, transforming growth factor (TGF)-β1, and CRP among others [4, 8].

A similar relationship has been proposed between quality of life and inflammation. A prospective observational study demonstrated a significant association between systemic inflammation and overall quality of life. When inflammation was stratified using the modified Glasgow Prognostic Score, the correlation was independent of performance status [9]. These associations are speculative, as the evidence to date is not sufficiently rigorous to draw firm conclusions. Nevertheless, malignancy-induced inflammation represents a potential therapeutic target to modify specific symptoms, symptom clusters, and overall quality of life.

Cannabidiol (CBD) is one of the major bioactive compounds in medicinal cannabis and is purported to have immunomodulatory properties. Unlike delta-9 tetrahydrocannabinol (THC), CBD is not psychoactive, which makes it desirable to many as a therapeutic option. Anecdotal data and some recent clinical evidence suggest that CBD may have a wide range of pharmacological properties including anxiolytic, antipsychotic, anti-oxidative, anticonvulsant, neuroprotective, and anti-inflammatory effects [10, 11]. CBD has been found to be an anti-inflammatory agent in many disease states, for example, in murine colitis, collagen-induced arthritis, neuroinflammation, and acute lung injury, where it dampened the production and release of the inflammatory mediators such as TNF-α, IL-1β, IFN-δ, IL-2, and NF-κB [10, 12]. There is no high quality clinical evidence to support the use of CBD as an anti-cancer agent, although it has shown in vitro anti-proliferative effects in breast [13], gastric [14], lung [15], prostate, and colorectal [16] cancer cells and in vivo antitumour effects in colorectal cancer [15, 17, 18].

Clinical trials are being undertaken to investigate the efficacy of CBD in patients with cancer and for the management of treatment-related complications. A recent Randomized controlled trial (RCT, MedCan-1) failed to show that CBD had any advantage over palliative care in managing symptoms related to advanced cancer [19]. Clinical studies considering the effect of CBD on inflammatory markers in adults with advanced cancer are lacking.

The present study sought to assess whether CBD would influence inflammatory markers in adults with advanced cancer in a randomised trial of CBD versus placebo. It was predicted that inflammatory marker expression would be significantly reduced in participants randomised to the CBD arm relative to the placebo arm. A positive result would justify a larger, adequately powered study of the anti-inflammatory effects of CBD in cancer patients.

The overall objectives of this study were:

(i) To determine whether participants randomised to CBD oil had reduced CRP levels between baseline and day 14 and between baseline and day 28 compared to those on placebo, in patients participating in MedCan-1, a randomised controlled trial of CBD vs placebo [19].
(ii) To determine whether CBD oil resulted in the reduction in levels of a panel of inflammatory markers, as measured in a subset of patients participating in MedCan-1.

## Methods

MedCan-1 (ANZCTR 26180001220257) was designed to determine whether CBD oil reduced total symptom burden in patients with advanced cancer to a greater extent than placebo and has been published previously [19]. In this trial, all participants were receiving palliative care. They were randomised to escalating doses of CBD oil (range 50 to 600 mg/day) or placebo oil as tolerated, over a 14-day period. Participants then continued at the patient-determined dose until day 28. The study of the potential anti-inflammatory effects of CBD (MedCan-Inflam) was undertaken as a sub-study of MedCan-1.

Participants provided written fully informed consent for both the parent and sub-study. Approval was obtained from the Mater Human Research Ethics Committee.

### Participants

A requirement of the parent study (MedCan-1) was the provision of a blood sample for CRP analysis at baseline, days 14, and 28. Additionally, a subset of participants (from those recruited through Mater Health Services only) were asked to consent to the inflammation sub-study (MedCan-Inflam) in which they were required to provide extra blood samples for analysis of inflammatory cytokines along with the required collection of blood for CRP measurement.

Participants remained on all their regular medications including anti-cancer therapy. Concomitant medication use was recorded and included in the analysis.

### Inflammatory marker analysis

As a convenience to participants, CRP assays were conducted by NATA accredited (https://nata.com.au/) pathology providers, including pathology departments at each of the 5 sites and commercial providers.

Blood for the inflammatory marker (cytokine) analysis (5 ml) was collected at each time point into BD Vacutainer® SST™ serum separation tubes, under standard conditions at the time of collection of other study bloods. Blood was allowed to clot at room temperature for 30 to 45 min followed by centrifugation at 1000 × *g* for 15 min at 4 °C and supernatant transfer into a clean tube, and a second centrifugation at 10,000 for 10 min at 4 °C. Sera were stored as undiluted aliquots in single-use polypropylene tubes at −80 °C until analysis.

Inflammatory marker analysis was conducted using the Bio-Plex 200 multiplex immunoassay system (Bio-Rad). Bio-Plex 200 allows a robust quantification of multiple cytokines for multiple patients in a 96-well plate in 3–4 h. Sample preparation was conducted using an automated, magnetic-bead wash station to ensure highest reproducibility standards. A 37-plex Bio-Plex Pro™ human inflammation panel 1 (Bio-Rad, Cat. #171-AL001M) was used to analyse pro- and anti-inflammatory serum cytokine levels following the manufacture’s recommendations. Briefly, the Bio-Plex 200 was calibrated prior to use. Serum samples were thawed and diluted in standard diluent HB at 1:4 factor. Sera, standards, blanks, and controls were assayed in duplicate with Bio-Plex human inflammation panel beads in 96-well plates. Assay quantitation was performed using lot-specific normalised standards (#54298167) and controls (#64310917) for all samples and cytokines.

### Sample size and statistics

The sample size of the primary study (MedCan-1) was calculated according to predicted improvement in symptom burden that has been shown from palliative care involvement in patients with advanced cancer [19].

The sub-study (Medcan-Inflam) was a convenience sample that was not powered to show any statistical difference. Anticipating an attrition rate of approximately 20% after 2 weeks, the primary analysis was planned for day 14. Within a sample of 30 participants, we anticipated approximately 15 would be receiving CBD and 15 placebo. Based on our pilot data, we anticipated the need to recruit 35 patients to have serum samples from 30 participants at day 14.

To avoid potential bias, unblinding did not occur until completion of the parent study.

Normally distributed data was summarised as mean (standard deviation, SD) and non-normally distributed data as median (inter-quartile range, IQR) or median (range). For normally distributed continuous data, groups were compared using a *t*-test and non-normally distributed data using Wilcoxon’s rank sum test. Categorical data were compared using Pearson’s chi-square or Fisher’s exact test.

The trajectory of inflammatory markers over time was evaluated using generalised estimating equations (GEE), to account for dropouts and missing data. The distributions of the inflammatory marker data were investigated using graphical (line graphs over time, histograms, and boxplots) and summary methods, and were almost invariably skewed to the right. A number of distributions and link functions were explored to select the best model. For most analyses, a normal distribution with log link was finally selected over a Gamma distribution with log link. Inferences were confirmed using simple difference methods (day 14 minus baseline, day 28 minus baseline) where appropriate. Data was analysed using SAS Version 9.4 (SAS Institute, Cary, NC, USA) and R (R Core Team (2021)) [20].

## Results

### CRP analysis

Characteristics of the participants in the parent study have been published previously [19]. All had advanced malignant disease, most commonly prostate, breast, colorectal, or gynaecological. Thirty-two of 71 (44.4%) of those in the placebo group were receiving chemotherapy or targeted therapy compared with 29/70 (41.4%) in the CBD group, *p* = 0.78. The majority of participants in both groups were receiving corticosteroids, either as part of a treatment regimen or for symptom control (40/71 (56.3%) of those in the CBD group and 47/72 (65.3%) of those in the placebo group, *p* = 0.27). At baseline, 4/71 (5.6%) participants on CBD were on non-steroidal anti-inflammatory medications compared with 10/72 (13.9%) of those on placebo, *p* = 0.16.

**Fig. 1:**
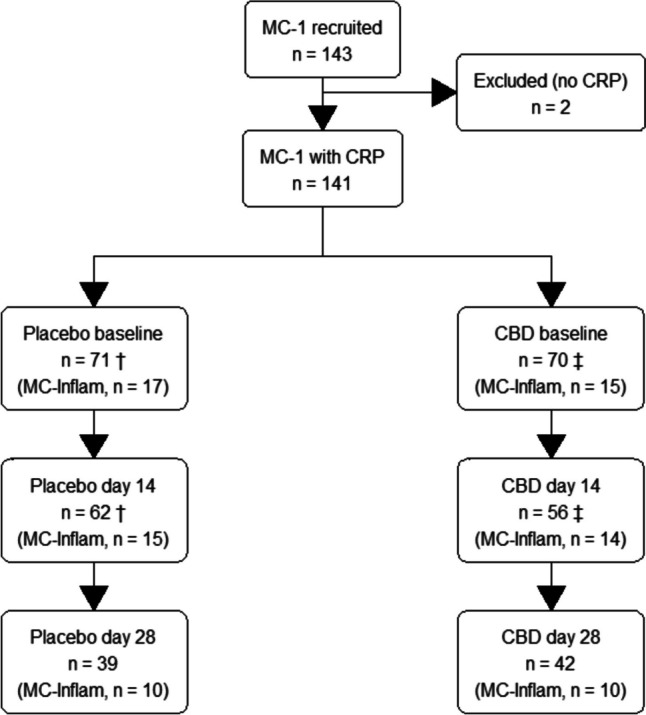
Flow of participants through the study. The single dagger (†) indicates CRP missing for one person; the double dagger (‡) indicates CRP missing for two people

The number of participants with CRP data available at each time point is shown in Fig. 1 (patient flow) and Table 1. CRP levels were available at baseline in 70/71 (99%) participants who received placebo and 68/70 (97%) of those who received CBD oil. Not all participants provided a CRP sample at all time points as shown in Fig. 1.

**Table 1:** CRP (median, range), mg/L at baseline, day 14, and day 28 by treatment arm

Time point	CRP (mg/L), CBD group	CRP (mg/L), placebo group	*p*-value
Baseline	18.5 (0.3–198) *n* = 68	15 (0.3–254) *n* = 70	0.81
Day 14	11.0 (0.3–240) *n* = 54	16 (0.3–196) *n* = 61	0.37
Day 28	7.9 (0.3–307) *n* = 42	8.1 (0.3–147) *n* = 39	0.69

**Fig. 2:**
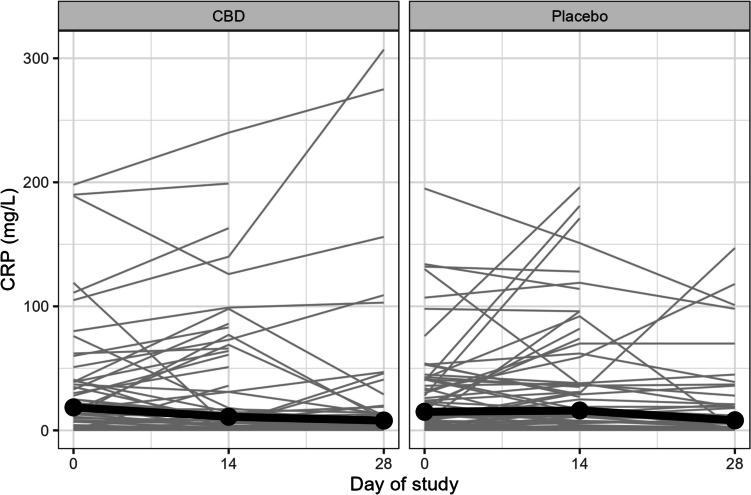
Line graph for CRP values from baseline to day 28 for 141 participants. Thick lines indicate the median CRP value and grey lines the values for each individual participant

The median CRP level at baseline was 17 mg/L (range 0.3–198) in the CBD group and 15 (0.3–254) in the placebo group (Table 1). There was no detectable difference in change in CRP levels from baseline between treatment arms at either day 14 or day 28. By day 14, the median (range) change in CRP was 0.0 (−116.1–65 mg/L, *n* = 53) in the CBD group compared with 0.2 (−93.0–143, *n* = 60) in the placebo group, *p* = 0.71. By day 28, the median (range) change in CRP from baseline was −1.0 (−114.1–202) mg/L, *n* = 41 in the CBD group and −0.15 (−94.0–140), *n* = 38, *p* = 0.93, in the placebo group (Fig. 2).

A confirmatory analysis using GEE, incorporating all available CRP measurements from all subjects from baseline to day 28, was consistent with these results, with a mean (95% CI) difference in change in CRP between the groups of 0.001 (−0.015–0.017) mg/L/day, *p* = 0.91.

The influence of corticosteroids on CRP was analysed (Supp Figure 1, Supp Table 1). There was no evidence of corticosteroid induced suppression of CRP levels.

### Cytokine analysis

A convenience sample of 33 MedCan-1 participants consented to the MedCan-Inflam sub-study. One did not complete baseline and was not randomised, three did not complete day 14, and a further 9 did not complete day 28. Patient characteristics are shown in Supplementary Table 2. The sub-study participants had a range of malignancies, the most common being prostate and colorectal cancer. Of those in the placebo group, 10/17 (59%) were receiving chemotherapy or targeted therapy compared with 6/15 (40%) of those in the CBD group, *p* = 0.29. Most participants in both groups were receiving corticosteroids, either as part of a treatment regimen or for symptom control (10 of 15 (66.7%) of those in the CBD group and 12/17 (70.6%) on placebo, *p* = 0.81). At baseline, one of the 15 participants on CBD was receiving non-steroidal anti-inflammatory medications compared with 2/17 in the placebo group, *p* = 1.0.

**Fig. 3:**
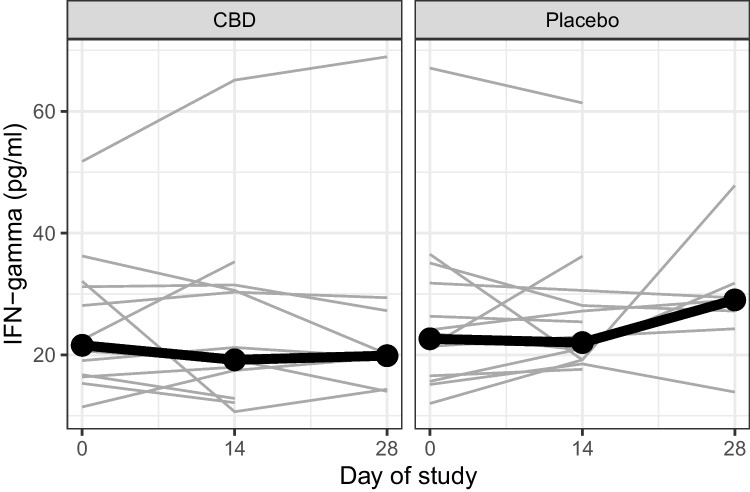
Line graph for IFN-gamma from baseline to day 28 for the 32 participants in the MedCan-Inflam sub-study. Thick lines indicate the median IFN-gamma value and grey lines the values for each individual participant

Cytokine analysis did not identify any difference in trajectory of any cytokine between the placebo and CBD groups (Table 2). An example of this showing the trajectory of IFN-gamma is shown in Fig. 3. Numbers of successful assays were insufficient to detect Hu IFN-a2, Hu IL-8, Hu IL-10,2,20,26,32,34, or 35, Hu MMP-1, and Hu LIGHT.

**Table 2:** Inflammatory cytokines over time

Cytokine	Placebo baseline* conc. [pg/ml] (no. participant) median (IQR)	Placebo day 14	Placebo day 28	CBD baseline	CBD day 14	CBD day 28	*p-*value*
April	(*n* = 16) 136,323.1 (108,398.4 to 166,673.8)	(*n* = 13) 137,493.1 (110,978.7 to 169,384.7)	(*n* = 10) 140,605.9 (121,820 to 175,522.4)	(*n* = 14) 175,095.2 (118,536.3 to 223,986)	(*n* = 13) 163,553.6 (142,096 to 212,624.1)	(*n* = 10) 176,663.6 (155,722 to 187,847.3)	0.73
Hu BAFF/TNFSF13B	(*n* = 16) 12,193.9 (7128.6 to 20,529.3)	(*n* = 13) 15,394.9 (7297.4 to 28,238)	(*n* = 9) 32,126.7 (5902.6 to 39,407)	(*n* = 14) 10,035.3 (4378.5 to 17,282.8)	(*n* = 13) 8530.7 (6814.3 to 29,895.5)	(*n* = 10) 7737 (5738.7 to 29,305.8)	0.35
Hu CD163	(*n* = 16) 55,580.6 (37,865.1 to 73,716.5)	(*n* = 13) 42,300 (29,117.9 to 59,592.4)	(*n* = 10) 37,679.5 (25,352.3 to 78,253.3)	(*n* = 14) 48,480.6 (25,618.1 to 64,939.1)	(*n* = 13) 42,328.3 (29,512.2 to 58,443.5)	(*n* = 10) 53,288.1 (40,546.2 to 79,296.8)	0.73
Hu CD30/TNFRSF8	(*n* = 16) 292.1 (148.1 to 383.4)	(*n* = 13) 303.2 (156.6 to 353.6)	(*n* = 9) 258. (152.5 to 397.8)	(*n* = 13) 217.6 (109.1 to 504.5)	(*n* = 11) 155.5 (136.2 to 460.9)	(*n* = 9) 423.8 (145 to 634.8)	0.50
Hu Chitinase 3-like 1	(*n* = 16) 9111.8 (6718.2 to 10,048.3)	(*n* = 13) 10,093.4 (6121.8 to 11,619.6)	(*n* = 10) 11,039.9 (4520.7 to 12,769.4)	(*n* = 14) 9362.2 (5682.8 to 11,506.4)	(*n* = 13) 7524 (5123.7 to 12,623.9)	(*n* = 10) 6675.6 (4483.3 to 12,420.5)	0.08
Hu IFN-b	(*n* = 14) 26.6 (19.7 to 30.4)	(*n* = 12) 21.2 (19.2 to 27.8)	(*n* = 8) 20.8 (16.6 to 27.1)	(*n* = 13) 24.1 (13.8 to 30.7)	(*n* = 12) 22.1 (16.9 to 28.2)	(*n* = 9) 22.2 (19.7 to 31)	0.37
Hu IFN-g	(*n* = 16) 22.6 (16.8 to 27.7)	(*n* = 13) 22 (19.2 to 27.2)	(*n* = 7) 29 (25.7 to 30.6)	(*n* = 12) 21.6 (16.6 to 31.4)	(*n* = 13) 19.2 (17.4 to 30.6)	(*n* = 8) 19.9 (18.3 to 27.8)	0.35
Hu IL-11	(*n* = 16) 9.3 (6.6 to 10.4)	(*n* = 13) 7.9 (6.4 to 9.7)	(*n* = 9) 8.7 (7 to 9.7)	(*n* = 13) 9 (5.8 to 10.7)	(*n* = 13) 9.6 (4.9 to 12.1)	(*n* = 9) 8.1 (7.1 to 12.7)	0.44
Hu IL-12	(*n* = 13) 47.8 (29.5 to 65.4)	(*n* = 10) 38.3 (28.3 to 49.8)	(*n* = 7) 37.7 (34.9 to 63.5)	(*n* = 9) 41.7 (28.2 to 106.2)	(*n* = 9) 35.2 (32 to 101)	(*n* = 7) 50.9 (35.3 to 84.3)	0.16
Hu IL-29/IFN-l1	(*n* = 11) 65.1 (59.6 to 71.9)	(*n* = 10) 71.3 (61.1 to 78.3)	(*n* = 6) 71.6 (64.3 to 81.4)	(*n* = 11) 70 (67.6 to 75.5)	(*n* = 11) 71.5 (60.4 to 84.9)	(*n* = 5) 80.5 (65.1 to 87.8)	0.99
Hu IL-6Ra	(*n* = 16) 3328.8 (3002.6 to 3867.9)	(*n* = 13) 3307.6 (2249 to 4240.7)	(*n* = 10) 3024.9 (2275.4 to 4080.7)	(*n* = 14) 4189.1 (3047.1 to 4961.8)	(*n* = 13) 3576.2 (3164.3 to 5232.2)	(*n* = 10) 4505.6 (2803.6 to 4728.4)	0.66†
Hu MMP-2	(*n* = 16) 5438.9 (1826.9 to 14,954.8)	(*n* = 12) 4631.7 (1970.3 to 13,070.9)	(*n* = 10) 4561.5 (2023.5 to 12,149.6)	(*n* = 13) 2740.6 (1757.2 to 9406)	(*n* = 10) 3892.9 (2376 to 10,442)	(*n* = 9) 10,117.7 (1878.8 to 11,996.6)	0.57
Hu MMP-3	(*n* = 16) 12,873.3 (8589 to 16,173.4)	(*n* = 11) 18,793.8 (10,573.9 to 24,857.5)	(*n* = 9) 16,306.1 (7750 to 23,430.6)	(*n* = 14) 18,433.8 (12,856.8 to 31,762.3)	(*n* = 13) 17,720.3 (16,010.4 to 31,268.5)	(*n* = 10) 16,819.6 (15,853 to 20,537.9)	0.18
Hu Osteocalcin	(*n* = 12) 1356.5 (869.8 to 3199.4)	(*n* = 12) 538 (314.6 to 1894.4)	(*n* = 7) 1202.7 (552.3 to 3719.2)	(*n* = 12) 934.9 (376.2 to 2774.4)	(*n* = 10) 1015.6 (431.6 to 2567)	(*n* = 9) 1178.6 (783.6 to 2348.2)	0.82
Hu Osteopontin	(*n* = 16) 5544.3 (3602.5 to 11,841.2)	(*n* = 12) 6352.8 (2892.4 to 7719.2)	(*n* = 10) 5871.6 (2589.3 to 12,840.9)	(*n* = 13) 7176.8 (3009.3 to 9435.1)	(*n* = 13) 6784.1 (1485.9 to 9404.6)	(*n* = 10) 7191.3 (2983.7 to 20,887.6)	0.93
Hu Pentraxin	(*n* = 16) 6269.4 (3996.3 to 8285.9)	(*n* = 13) 4797.4 (3525.2 to 10,980.4)	(*n* = 10) 5768.3 (3207.7 to 8664.6)	(*n* = 14) 3115.9 (2607.2 to 11,801.7)	(*n* = 13) 4751.2 (2852.1 to 13,028.8)	(*n* = 10) 3839.1 (2133.8 to 15,217.9)	0.74
Hu TNF-R1	(*n* = 15) 659.5 (277.1 to 818.2)	(*n* = 11) 507.2 (339.8 to 708)	(*n* = 8) 565.2 (280.5 to 869.6)	(*n* = 14) 417.5 (329.9 to 1084.9)	(*n* = 13) 435.6 (302.1 to 820.9)	(*n* = 10) 745.9 (376.4 to 1174.1)	0.86
Hu TNF-R2	(*n* = 16) 204.7 (159.12 to 431.4)	(*n* = 13) 170.2 (143.7 to 407.3)	(*n* = 9) 203.9 (187.6 to 233.5)	(*n* = 14) 212.2 (156.7 to 399.1)	(*n* = 13) 222.1 (181.2 to 377.2)	(*n* = 10) 336.8 (208.9 to 543.4)	0.45
Hu TSLP	(*n* = 16) 27.1 (17.6 to 35.4)	(*n* = 12) 26.1 (20 to 32.5)	(*n* = 9) 27.6 (26.4 to 29.9)	(*n* = 14) 27.4 (18.3 to 56.3)	(*n* = 13) 33.1 (17.9 to 55)	(*n* = 10) 32.7 (16 to 39.3)	0.51
Hu TWEAK/TNFSF12	(*n* = 16) 146.7 (90.9 to 295.5)	(*n* = 13) 97.7 (75.1 to 351.8)	(*n* = 9) 111.7 (94.3 to 202.4)	(*n* = 13) 201 (86.8 to 251.9)	(*n* = 11) 144.3 (103.6 to 393.1)	(*n* = 9) 165.3 (120.7 to 359.9)	0.24
Hu gp130	(*n* = 16) 22,410.6 (11,938.4 to 30,716.7)	(*n* = 13) 13,007.2 (11,605.1 to 25,924.8)	(*n* = 10) 13,668.5 (11,772.9 to 28,182.7)	(*n* = 14) 14,461.5 (11,499.6 to 26,121.6)	(*n* = 13) 15,392.9 (11,462.8 to 19,021.4)	(*n* = 10) 18,528 (12,652.6 to 24,687.9)	0.56

*p-value for difference in change (trajectory over time)

†Normal distribution with identity link

## Discussion

There has been considerable pre-clinical work suggesting that CBD has an anti-inflammatory effect [12] and this has been postulated as a reason why many people report pain relief when taking cannabis. This was a pilot study to assess whether CBD reduces inflammatory cytokines in patients with advanced cancer. CBD administered as part of a dose-escalating protocol to patients with advanced cancer did not reduce inflammatory markers as compared to placebo. The median dose (400 mg/day) selected by participants was determined by tolerance and is similar to that used in many therapeutic studies published in the literature [21]. Furthermore, as reported elsewhere, CBD did not appear to have a clinically relevant anti-inflammatory effect in that it did not reduce pain or overall symptom burden any more than placebo in the primary RCT [19].

Median CRP at baseline was of sufficient magnitude to be susceptible to any anti-inflammatory effect of CBD. Minor discrepancies between the lower limit of “normal” CRP between laboratories were allowed for by the calculation of median change in CRP levels as opposed to absolute change.

The 37-plex Bio-Plex Pro™ human inflammation panel allowed for the detection of a wide range of pro- and anti-inflammatory markers and has been used previously to reflect various pathophysiological conditions and inflammation status in patients [22].

These results are consistent with a recent randomised controlled trial in which CBD was tested against placebo for the management of symptoms of coronavirus in which a range of inflammatory cytokines were tested and found not to be impacted by cannabis use [23]. The baseline CRP levels in this study were relatively low, however. Clinical trials of cannabis in patients with inflammatory bowel disease have also failed to show a reduction on inflammatory markers [24].

The lack of benefit on average does not preclude benefit in select cases. There may be certain characteristics in either the patient or the cancer which predict anti-inflammatory response to CBD. Future investigators may consider restricting their population to patients with those cancers suggested to elicit a more pronounced systemic inflammatory response [25].

Around one-half of all patients in this study were receiving anti-cancer therapy and most were on corticosteroids, but there was no significant difference in proportions of patients on these therapies between the treatment groups.

This study utilised a pure synthetic CBD product. It is possible that any anti-inflammatory effect of cannabinoids relies on the presence of terpenes and other components contained plant-based products through an “entourage” effect. Similarly, there is some existing research suggesting an anti-inflammatory effect for combined THC and CBD in animals but no studies to date in an adult cancer population. We are planning a similar study in patients with advanced cancer administered a THC/CBD (1:20) oral suspension or placebo as part of a larger study assessing the benefit of this product for reducing total symptom burden.

In summary, we found no evidence to suggest that CBD has an anti-inflammatory role in patients with advanced cancer and no indication to proceed with this agent to a larger, properly powered, prospective study. The potential role if any of THC deserves further investigation.

### Supplementary information

ESM 1 (PDF 293 kb)

## Data Availability

All raw data are available on request from the authors.
