# Supplementary material for: Exploring potential anti-inflammatory effects of medicinal cannabis
Source: Support Care Cancer. 2023 Oct 14;31(11):629. doi: 10.1007/s00520-023-08069-8 (PMC10576726; doi:10.1007/s00520-023-08069-8)
Supplement: Supplementary file 1 — (PDF 293 kb) [file 520_2023_8069_MOESM1_ESM.pdf]

Exploring potential anti-inflammatory effects of medicinal cannabis

Taylan Gurgenci[1,2]*, Gregor Kijanka[3]*, Ristan Greer [2,4], Georgie Huggett[1], Phillip Good [1,2,5], Md Moniruzzaman[2,6], Janet Hardy[1,2]

*joint first authors

[1]Department Palliative and Supportive Care, Mater Health Services, South Brisbane, Queensland, Australia

[2]Mater Research Institute - University of Queensland, Brisbane, Queensland, Australia

[3]Queensland Micro- and Nanotechnology Centre, Griffith University

[4]Torus Research, Brisbane, Queensland, Australia

[5]Department of Palliative Care, St Vincent's Private Hospital Brisbane, Brisbane, Queensland, Australia

[6]School of Pharmacy, The University of Queensland, Brisbane, Australia

Supplementary Figure 1. Line graphs for CRP from baseline to day 28 by treatment arm and corticosteroid exposure.

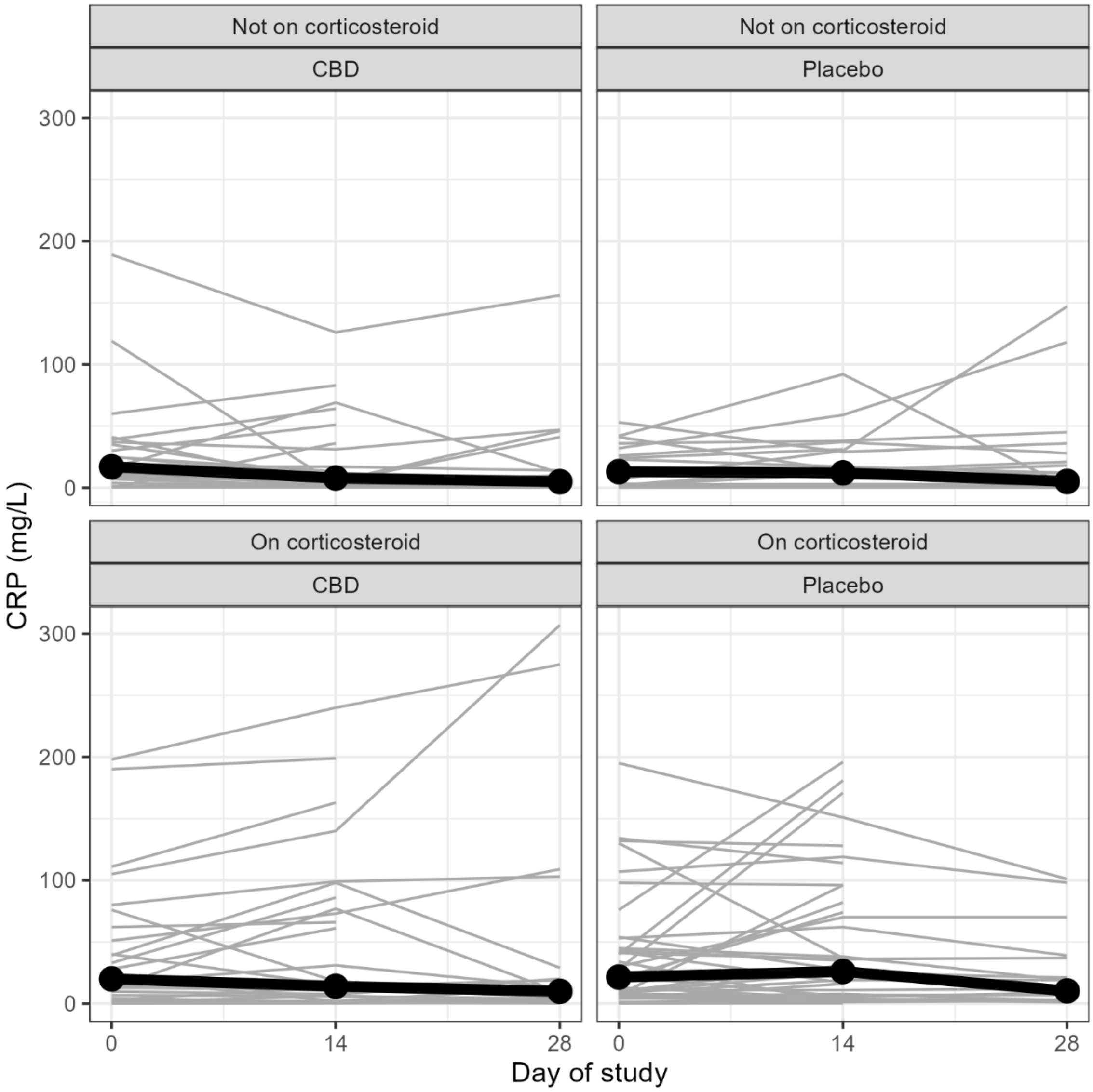

Thick lines indicate the median CRP and grey lines the values for each individual participant.

Supplementary table Table 1. CRP (median (range)), mg/L at baseline, day 14 and day 28 by treatment arm and corticosteroid exposure*.

time point	CRP (mg/L), CBD group	CRP (mg/L), Placebo group	p-value
baseline: not on corticosteroids	17.0 (0.3 – 189), n=29	13.0 (0.3 – 53), n=24	0.21
baseline: on corticosteroids	20.0 (0.3 – 198), n=39	21.5 (0.3 – 254), n=46	0.74
day 14: not on corticosteroids	7.8 (0.4 – 126), n=25	12.0 (0.3 – 92), n=23	0.75
day 14: on corticosteroids	14.0 (0.3 – 240), n=29	26.0 (0.3 – 196), n=38	0.65
day 28: not on corticosteroids	4.9 (0.4 – 156), n=17	5.1 (0.3 – 147), n=21	0.68
day 28: on corticosteroids	9.9 (0.3 – 307), n=25	10.3 (1.0 – 101, n=18	0.91

*Excludes those with missing CRC data

Supplementary Table 2. Participant characteristics (MedCan-Inflam)

	CBD<br>n=15	Placebo<br>n=17
Age (median, IQR)	69.0 (66.5 to 79.5)	73.0 (62.0 to 79.0)
Gender M/F (%M)	10/5 (66.7%)	6/11 (35.3%)
Primary Cancer		
Brain	1 (6.7)	0 (0.0)
Breast	0 (0.0)	5 (29.4)
Colorectal	2 (13.3)	3 (17.6)
Gynecological	1 (6.7)	2 (11.8)
Haematological	1 (6.7)	0 (0.0)
LiverHCC	1 (6.7)	0 (0.0)
Lung	2 (13.3)	0 (0.0)
Lung,Colorectal	1 (6.7)	0 (0.0)
Pancreas	0 (0.0)	1 (5.9)
Prostate	5 (33.3)	5 (29.4)
Prostate,Haematological	0 (0.0)	1 (5.9)
Urothelial	1 (6.7)	0 (0.0)
